# Origin, Potential Therapeutic Targets and Treatment for Coronavirus Disease (COVID-19)

**DOI:** 10.3390/pathogens9040307

**Published:** 2020-04-22

**Authors:** Muhammad Shahid Nadeem, Mazin A. Zamzami, Hani Choudhry, Bibi Nazia Murtaza, Imran Kazmi, Habib Ahmad, Abdul Rauf Shakoori

**Affiliations:** 1 Department of Biochemistry, Faculty of Science, King Abdulaziz University, Jeddah 21589, Saudi Arabia; mzamzami@kau.edu.sa (M.A.Z.); hchoudhry@kau.edu.sa (H.C.); ikazmi@kau.edu.sa (I.K.); 2 Department of Microbiology, Abbottabad University of Science and Technology, Abbottabad 22010, Pakistan; nazia.murtaza@gmail.com; 3 Department of Genetics, Hazara University Garden Campus, Mansehra 21300, Pakistan; drhahmad@gmail.com; 4 School of Biological Sciences, University of the Punjab, Lahore 54000, Pakistan; arshakoori.sbs@pu.edu.pk

**Keywords:** COVID-19, SARS-CoV-2, origin, pathogenesis, therapeutics, challenges

## Abstract

The ongoing episode of coronavirus disease 19 (COVID-19) has imposed a serious threat to global health and the world economy. The disease has rapidly acquired a pandemic status affecting almost all populated areas of the planet. The causative agent of COVID-19 is a novel coronavirus known as SARS-CoV-2. The virus has an approximate 30 kb single-stranded positive-sense RNA genome, which is 74.5% to 99% identical to that of SARS-CoV, CoV-pangolin, and the coronavirus the from horseshoe bat. According to available information, SARS-CoV-2 is inferred to be a recombinant virus that originated from bats and was transmitted to humans, possibly using the pangolin as the intermediate host. The interaction of the SARS-CoV-2 spike protein with the human ACE2 (angiotensin-converting enzyme 2) receptor, and its subsequent cleavage by serine protease and fusion, are the main events in the pathophysiology. The serine protease inhibitors, spike protein-based vaccines, or ACE2 blockers may have therapeutic potential in the near future. At present, no vaccine is available against COVID-19. The disease is being treated with antiviral, antimalarial, anti-inflammatory, herbal medicines, and active plasma antibodies. In this context, the present review article provides a cumulative account of the recent information regarding the viral characteristics, potential therapeutic targets, treatment options, and prospective research questions.

## 1. Introduction

The outbreak of a novel respiratory syndrome, referred to as coronavirus disease 2019 (COVID-19), was first recognized in Wuhan, China, in December 2019. The causative agent for this deadly condition is a coronavirus known as SARS-CoV-2. COVID-19 is demonstrated by fever, dry cough, persistent pressure in the chest, and shortness of breath [1,2]. Sneezing, runny nose, and symptoms similar to the common cold are observed in only 5% of patients. About 2% to 10% of patients have shown diarrhea like symptoms [3,4]. The mortality rate of COVID-19 is 4.5% to 6%, which is less than that of SARS (severe acute respiratory syndrome), which has a mortality rate of 9.6%, and less than that of MERS (Middle East respiratory syndrome), up to 34.4% deaths. Individuals already suffering from cardiovascular disease, hypertension, respiratory disease, or diabetes are at a high risk of mortality. Age and gender-specific variations in the death rate have also been reported [5]. The disease became pandemic within a few months of its emergence, indicating a high transmission ability as compared with SARS and MERS [6,7,8]. COVID-19 lasts up to 6 weeks depending upon the individual’s immunity and the disease intensity. A variable incubation period has been reported for the infection to establish completely; a second exposure to the viral inoculum may decrease the incubation time [7]. An incubation time of 3 to 27 days (average 14 days) has been reported by different sources [6,9,10]. This incubation period is considerably longer than that required by SARS or MERS [11,12]. SARS-CoV-2 has an airborne route of transmission, whereby small aerosols spread in the surrounding air by the coughing and sneezing of infected individuals. These fine airborne droplets, harboring viral particles, can be directly inhaled by nearby healthy individuals [13]. The viral particles can stick to the fingertips and invade the healthy individuals by contact of contaminated hands with the nose, eyes, or mouth. Hence, hand hygiene is an expedient precaution to reduce SARS-CoV-2 transmission [14]. There is no evidence for the sexual transmission of SARS-CoV-2; however, the possibility of fecal transmission has been reported [15,16]. The infection mechanism of both the SARS and COVID-19 viruses involve an interaction with the angiotensin-converting enzyme 2 (ACE2) and cleavage of the viral spike protein by a serine protease [17,18]. Hence, a similar set of therapeutic targets can be the subject of prospective investigations. The present review article provides a cumulative account of recent information on the origin of the virus, its characteristics, and the potential therapeutics for COVID-19.

## 2. Origin, Transmission and Diagnosis of SARS-CoV-2

**Figure 1:**
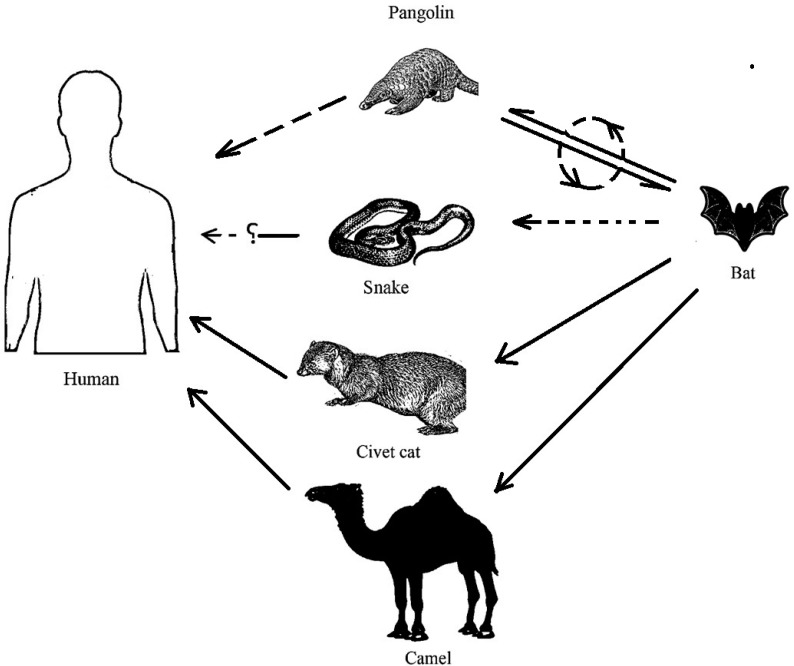
Intermediate hosts for the SARS virus (civet cat), the MERS virus (camel), and the possible intermediate hosts for SARS-CoV-2 (pangolin or snake). The dotted lines indicate intermediate hosts under investigation (adopted and modified from literature) [33,34,43].

Coronaviruses, first discovered in the 1960s, are found in birds and mammals, especially in bats, cats, camels, and rats [19]. The causative agent of COVID-19 (SARS-CoV-2) belongs to the genus *β-Coronavirus*, family Coronaviridae, and order Nidovirales. A similar human coronavirus was found to be responsible for SARS in 2002 and 2003. The virus responsible for COVID-19 has a single-stranded positive-sense RNA genome of about 30 kb, which has 74% to 99% identity with that of the coronavirus from the pangolin (*Manis javanica*) and horseshoe bat (*Rhinolophus sinicus*) (Bat-CoVRaTG13), respectively [2,20,21,22,23]. Bats have been reported as being the rich source of coronaviruses [24,25], although only a few of these coronaviruses can infect humans [26,27]. According to the literature, the SARS and MERS viruses have zoonotic transmission, originating from bats using palm civets and camels, respectively, as the intermediate hosts [28,29,30,31,32]. The recent reports have suggested that SARS-CoV-2 is a modified coronavirus of bat origin [22,32], which came to humans as a result of zoonotic transmission [33,34]. A coronavirus identified from the Malayan pangolin has been shown to have a 99% similarity with SARS-CoV-2. The receptor-binding domain (RBD) of pangolin-CoV has only a one amino acid difference with that of SARS-CoV-2; the infected pangolins exhibit pathological symptoms similar to humans suffering from COVID-19, and their blood circulating antibodies can react with the spike protein of SARS-CoV-2 [35,36]. Although the RaTG13 coronavirus isolated from bat has about 96% identity with SARS-CoV-2, its RBD is different from that of the later, exhibiting a low binding ability to the human ACE2 [37]. However, the RBD of the S-protein from pangolin-CoV is highly similar to that of SARS-CoV-2, six residues critical for receptor binding being identical in both [38]. A comparative analysis of genetic data available to date has suggested that SARS-CoV-2 originated by the recombination of pangolin-CoV and the bat-CoV-RaTG13-like virus [35,39,40,41]. Based on this information, the pangolin is considered to be one of the possible intermediate hosts between bat and human. Snakes, minks, and turtles are also being investigated as the potential intermediate hosts [42,43] (Figure 1). Five out of the six critical amino acid residues comprising the RBD of the S-protein from SARS-CoV and SARS-CoV-2 are different, contradicting the theories about the laboratory origin of SARS-CoV-2 by the manipulation of SARS or MERS like viruses [43]. Studies based on the analysis of *N*, *S*, and *ORF1a/1b* genes have shown conserved sequences suggesting that SARS-CoV-2 is an animal virus, which was transmitted to humans by undergoing evolutionary adaptations [22,44]. The SARS infection from 2003, also involved zoonotic transmission of the virus to humans. Hence, further studies are required to confirm the intermediate hosts of coronaviruses to control zoonotic transmission and avoid the outbreak of such viral infections in the future [28].

On 2 March 2020, WHO published a PCR based detection method for SARS-CoV-2. The procedure could detect the virus in the blood, sputum, and nasopharyngeal swab [45,46]. Noncontrast chest CT (computed tomography) can also be used for the diagnosis of viral pneumonia. However, CT scans can be negative in the case of COVID-19 [47]. On the other hand, patients with negative RT-PCR test results can show pneumonia-like symptoms on a CT scan [48]. In a comparative study, the sensitivity of a chest CT was found to be 98%, whereas the sensitivity of the PCR test was only 71% [49,50]. RT-PCR based diagnosis also gave false-positive results [51]. Low viral load, inefficient sampling, poor sample storage or processing conditions, along with a lack of specific primers due to the high rate of mutations in RNA viruses, are some of the apparent factors for the poor sensitivity of PCR based diagnoses. Recently, some parallel procedures have also been reported for the diagnosis of COVID-19. One of these procedures is loop-mediated isothermal amplification (LAMP), which is a faster single-step procedure, having >95% sensitivity [52,53]. Further modifications of LAMP-based procedures have been reported, which can reduce the testing time with minimum equipment requirements [54,55]. To develop serological procedures, IgA and IgM have been evaluated against SARS-CoV-2 by immunofluorescence assays [56,57,58]. However, further refining of RT-PCR and the serological procedures are required to improve the sensitivity and specificity.

### 2.1. SARS-CoV-2 vs. SARS-CoV—A Brief Comparison

SARS became epidemic in many countries around the world in 2002 and 2003. The disease had many symptoms similar to those of COVID-19. However, SARS-CoV-2 and SARS-CoV have shown differences, as well as similarities, in their genomic composition, incubation time, and infection mechanisms. A set of affinities has been tabulated that can help us to establish the correlation between the two viruses (Table 1).

### 2.2. Potential Therapeutics and Treatment for COVID-19

**Figure 2:**
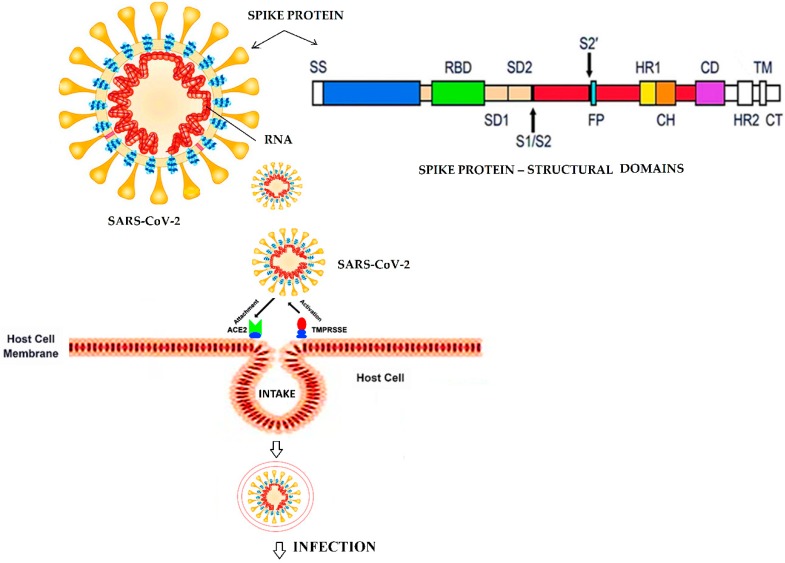
The interaction of the viral S-protein with ACE2, its subsequent activation by protease (TMPRSS2), and its viral entry into the cell. The schematic primary structure of the SARS-CoV-2 spike protein is elaborated indicating the major domains. SS—signal sequence, RBD—receptor binding domain, RBD subdomains 1 and 2—SD1 and SD2, S1/S2—the protease cleavage site, S2′—the protease restriction site indicated by the arrows, FP—fusion peptide, HR1 and HR2—heptad repeats 1 and 2, CH—central helix, CD—connector domain, TM—transmembrane domain, and CT—cytoplasmic tail. (The schematic was adopted and modified from [87,88].)

The intra- or inter-species transmission of β-coronaviruses (CoVs) requires a viral interaction with the host cell receptors, and the subsequent invasion of the host cells [85]. The genome of the coronavirus codes for a surface glycoprotein, known as a “spike” protein (S-protein), that specifically binds to the host cellular receptors to initiate the infection process. In fact, the spike protein performs a “key” like function to “unlock” the door and facilitate the cellular entry of a coronavirus. Studies based on 3D models of spike proteins from the SARS-CoV and SARS-CoV-2 viruses, have shown a considerable overall similarity [34,86]. The cryo-EM structure of the SARS-CoV-2 S-protein has also been reported [87] (Figure 2). The overall structure of the S-protein consists of several functional domains. The RBD, fusion domain (FD), and the S2 cleavage site could be critical for future studies to develop therapeutic strategies [87]. The protein exhibits a high binding affinity with ACE2, as represented by a low dissociation constant value (Kd ⁓ 15 nM). The receptor binding affinity of the SARS-CoV-2 S-protein is 10 times higher than that of the SARS-CoV S-protein [37,87,88,89,90]. Furthermore, studies using human, pig, and civet cell lines have allowed SARS-CoV-2 infection and replication, indicating that the virus makes use of the ACE2 receptor for infection [22,91,92,93]. ACE2 is cleaved by a protease (TMPRSS2) in order to activate virus entry. This can be inhibited by protease inhibitors such as camostat mesylate [92]. ACE2 is highly expressed in the lungs; a vast surface area makes the lung tissue highly susceptible to SARS-CoV-2 infection [94]. In addition to the lungs, the ACE2 receptor is also expressed in the endothelial cells of intestine, kidney, and heart cells [95].

According to recent information, the glutamine at the amino acid position 394 in the receptor-binding protein of SARS-CoV-2 that corresponds to the residue 479 in SARS-CoV, is recognized by lysine 31 residue in the human ACE2 receptor [90]. An interaction of polar residues in the ectodomain of ACE2 with the receptor binding domain of SARS-CoV-2 spike protein has been reported [96]. Downstream interaction and the invasion process include the cleavage of the spike by a serine protease at the “S2” domain (Figure 2) [97], followed by the interaction of the virus S-protein fusion domain with the host cell plasma membrane. Virus entry takes place either by fusion with the plasma membrane or by endocytosis, and the subsequent fusion of membranes in endosomes [98]. In addition to virus–plasma membrane fusion, SARS-CoV can adopt clathrin-dependent endocytosis [27]. Once inside the host cell, the viral RNA is translated in the cytoplasm producing polyproteins and structural proteins. After translation, the replication of the genome occurs [99]. New virus particles are formed in the membranes of the Golgi apparatus and the endoplasmic reticulum, after which the vesicles harboring the viral particles are fused with plasma membranes for the release of the virus [100,101]. Because SARS-CoV-2, in binding with ACE2, is dependent on an interaction and the cleavage of the spike protein by a serine protease, a spike protein-based vaccine or serine protease specific inhibitors could be potential therapies against SARS-CoV-2 infection [102]. The comparative homology studies of SARS-CoV-2 proteins (S, N, M, and E proteins) with those from SARS and MERS viruses, have suggested some targets for vaccine development [103]. Polyclonal antibodies raised against SARS-CoV are found to prevent a spike-mediated host cell invasion of SARS-CoV-2 [104]. Recently, 1.3 billion potential protease blockers have been investigated by molecular docking studies. Many of these molecules can be evaluated in wet labs in the near future [105]. ACE2 blockers can be another option to avoid the infection [106]. Similarly, there are some molecules including GSK1838705A, KT203, KT185, and BMS195614 that have strong binding affinities with RBD of the viral S-protein [107]. These molecules can help to control rapid infections by engaging the virus at entry points [107].

Currently, a tremendous amount of research is in progress to develop a vaccine against COVID-19. However, vaccine development is time consuming process, and the newly introduced vaccine will require several safety evaluations [4]. According to estimates, a vaccine against COVID-19 may take more than a year to become available [108]. Even after the preparation of an effective vaccine, under the present pandemic situation, human trials will be a big challenge for researchers. At present COVID-19 is being treated with some broad-spectrum antiviral drugs including remdesivir, favipiravir, and Chinese herbal medicine [101,109]. In vitro studies have shown that chloroquine and remdesivir are effective against SARS-CoV-2 [73,74,75]. Chloroquine phosphate has shown treatment efficacy and safety against SARS-CoV-2 associated pneumonia; these findings are based on multiple trials in hospitals in China [76]. Application of an anti-inflammatory drug such as baricitinib, together with an antiviral drug, has also been recommended to treat COVID-19 [110]. High doses of ascorbic acid (vitamin C) are suggested for the prevention of the COVID-19 disease [111]. Type I interferon can inhibit viral replication. According to studies, interferon β could inhibit the replication of SARS-CoV [95,112]. However, its efficacy against SARS-CoV-2 needs further investigation. The use of convalescent plasma for the treatment of COVID-19 has been suggested. However, the absence of multiple trials on a large scale, and the concern that antibodies may demonstrate donor dependent titters and specificities, are the main deficits of convalescent plasma therapy [113].

## 3. Prospective Challenges and Research Questions

There are several challenges in the management and control of coronaviruses. A wide range of coronaviruses with a highly mutable single-stranded RNA genome are found [20,21] in many mammalian and avian sources [19] that closely interact with each other, as well as with humans. The long-term viability of coronaviruses in airborne aerosols, and on daily utensils composed of plastics, stainless steel, and cardboard, enhance the chances of transmission of infection between individuals and species [8,81,114]. The recurrence of SARS-CoV-2 has been reported in convalescence times [115], which can make the treatment more difficult and increase the chance of complications. The interventions into the ACE2 binding abilities of SARS-CoV-2 and similar viruses, by the inhibition of the corresponding serine protease [106], can be an area of investigation. Easy, highly reliable, and early-stage diagnosis procedures are still required as a challenge for biomedical researchers [52,53]. The presence of SARS-CoV-2 in stool samples of infected individuals raises the question about the fecal-oral transmission of the disease [10]. Recently, the viability of SARS-CoV and SARS-CoV-2 has been described (115); however, the genetic factors behind the long-term survival of these coronaviruses need further investigation [81]. SARS-CoV has shown low stability at higher temperatures and at specific air humidity levels [9,116]; the effect of temperature and other environmental factors on the viability of SARS-CoV-2 are unclear. Inactivation of coronaviruses by disinfectants, such as 60% to 70% ethanol or 0.1% sodium hypochlorite, is well established [117]. The efficacy and specificity of antiviral and antimalarial drugs being used in the treatment of COVID-19 need further clinical trials.

## 4. Conclusions

The ongoing COVID-19 outbreak that emerged from Wuhan, China, has acquired pandemic status. The causative agent of COVID-19 is a modified coronavirus, known as SARS-CoV-2 that has similarities with the coronaviruses responsible for SARS, MERS, and those identified as coming from various animals including the pangolin and bat. SARS-CoV-2 specific vaccine development, and the application of highly specific antiviral medicines, require time and investigation. The health management authorities have a major focus on known preventive measures for viral infections. The situation demands keen surveillance, and the development of early diagnostic and better treatment options. The present report provides an insight into the characteristics of the pathogen, its mode of infection, its potential targets, along with future research questions.

## Figures and Tables

**Table 1:** Comparative analysis of COVID-19 and SARS with reference to their corresponding causative agents, symptoms, origins, and therapeutics.

Sr. No.	COVID-19 (SARS-CoV-2)	SARS (SARS-CoV)	References
1	COVID-19 is represented by pneumonia-like symptoms, fever, cough, or diarrhea. The outbreak of disease was recorded in December 2019, in China.	SARS showed many symptoms similar to that of COVID-19. The outbreak was detected in November 2002 (winter), in China.	[44,59,60,61]
2	To date, the mortality rate of COVID-19 is 4.5% to 5.5%. There are more than 1 million reported infections and 50,000 deaths (as recorded on 3 April 2020).	The mortality rate was between 9.6% to 21%. It was restricted to 8437 individuals and 813 deaths.	[6,7,62]
3	The virus needs a longer incubation time (average 14 days) to represent COVID-19 symptoms.	The virus needed a relatively short incubation time (1–4 days) to exhibit symptoms.	[11,63]
4	In COVID-19, the infection ratio between males and females is 2.7:1, indicating that the disease is more prevalent among males. Old aged people also have a high mortality rate.	The male to female ratio was 1:1.25; more prevalent in females. There was a higher death rate in old aged patients.	[3,64]
5	SARS-CoV-2 has a potential origin from bats, and it is suspected to have a zoonotic transmission involving an unclear intermediate host. The pangolin is considered as a probable intermediate host; snakes, minks, and turtles are also being investigated.	SARS-CoV originated from bats. It has zoonotic transmission via the civet cat as the intermediate host.	[65,66,67,68]
6	Several diagnostic tools including RT-PCR, chest CT, LAMP, etc., have been applied to detect COVID-19. However, the efficacy and sensitivity of these methods is still under investigation.	SARS was efficiently diagnosed by RT-PCR.	[53,69,70,71]
7	COVID-19 is being treated by antiviral, antimalarial, and anti-inflammatory medicine. It is also being treated by the transfusion of active plasma antibodies into the blood circulation of infected patients.	SARS was treated by antiviral drugs including ribavirin and interferon.	[72,73,74,75,76]
8	Low temperature is more suitable for SARS-CoV-2 viability and pathogenicity.	Infection ability and viability were temperature dependent.	[11,77]
9	COVID-19 infections have spread to over 99.8% of the global populated area.	SARS was restricted to 29 countries in the world.	[78,79,80]
10	SARS-CoV-2 remains viable in aerosols, and on plastic and steel surfaces, for a considerable time. The virus is not viable on copper after 4 h, nor on cardboard after 24 h.	SARS-CoV was found viable in aerosols for 3 h, on plastic for 72 h, and on steel for 48 h. It was not viable on copper after 8 h, nor on cardboard after 8 h.	[81]
11	The mechanism of the SARS-CoV-2 infection transmission is similar to that of the influenza virus.	The transmission mode of SARS-CoV is not similar to that of the influenza virus.	[46,82,83]
12	Recent reports advocate the asymptomatic transmission of SARS-CoV-2.	Asymptomatic transmission of SARS-CoV has also been reported.	[9,46,64,84]
13	Six amino acids, Leu455, Phe486, Gln493, Ser494, Asn501, and Tyr505 are critical in ACE2 binding to the domain of SARS-CoV-2.	The corresponding amino acids in SAR-CoV are: Tyr442, Leu472, Asn479, Asp480, Thr487, and Tyr4911. This indicates that five out of six amino acids are different to that of SARS-CoV-2.	[37,43]
